# Spatio‐temporal expression dynamics differ between homologues of flowering time genes in the allopolyploid *Brassica napus*

**DOI:** 10.1111/tpj.14020

**Published:** 2018-08-24

**Authors:** D. Marc Jones, Rachel Wells, Nick Pullen, Martin Trick, Judith A. Irwin, Richard J. Morris

**Affiliations:** ^1^ Crop Genetics John Innes Centre Norwich Research Park Norwich NR4 7UH UK; ^2^ Computational and Systems Biology John Innes Centre Norwich Research Park Norwich NR4 7UH UK

**Keywords:** *Brassica napus*, gene duplication, flowering time, polyploid, regulation, time series

## Abstract

Polyploidy is a recurrent feature of eukaryotic evolution and has been linked to increases in complexity, adaptive radiation and speciation. Within angiosperms such events have occurred repeatedly in many plant lineages. Here we investigate the retention and spatio‐temporal expression dynamics of duplicated genes predicted to regulate the floral transition in *Brassica napus* (oilseed rape, OSR). We show that flowering time genes are preferentially retained relative to other genes in the OSR genome. Using a transcriptome time series in two tissues (leaf and shoot apex) across development we show that 67% of these retained flowering time genes are expressed. Furthermore, between 64% (leaf) and 74% (shoot apex) of the retained gene homologues show diverged expression patterns relative to each other across development, suggesting neo‐ or subfunctionalization. A case study of homologues of the shoot meristem identity gene *TFL1* reveals differences in *cis*‐regulatory elements that could explain this divergence. Such differences in the expression dynamics of duplicated genes highlight the challenges involved in translating gene regulatory networks from diploid model systems to more complex polyploid crop species.

## Significance Statement

Many economically important crops exhibit extensive gene duplication due to recent or ancestral polyploidisation. This study demonstrates that flowering time genes have been preferentially retained in *Brassica napus* and that significant differences in the spatio‐temporal expression dynamics occur between gene homologues.

## Introduction

Many economically important crops exhibit extensive gene duplication as a result of repeated recent or ancestral rounds of polyploidy (Renny‐Byfield and Wendel, 2014), examples include wheat (*Triticum aestivum*) (International Wheat Genome Sequencing Consortium, 2014), cotton (*Gossypium hirsutum*) (Li *et al*., 2015) and oilseed rape (OSR, *Brassica napus*) (Chalhoub *et al*., 2014). The presence of multiple copies of a gene relaxes natural and artificial selective pressures on any one individual copy, facilitating the emergence of novel gene functions (Conant and Wolfe, 2008). The resulting increase in variation can be exploited to breed crop varieties with desirable phenotypes (Otto, 2007). The presence of multiple orthologues, however, complicates efforts to translate knowledge of gene function, and especially of gene regulatory networks, from model to crop species. This is a consequence of not knowing which orthologue, if any, retains the same function as the corresponding gene in the model species, whether ancestral functions have been partitioned between them, or if a novel function has been acquired (Lynch and Conery, 2000).

The genus *Brassica* contains several diploid crop species derived from ancestors that underwent a whole‐genome triplication event 5–28 million years ago (Lysak *et al*., 2005; Beilstein *et al*., 2010; Wang *et al*., 2011). Oilseed rape is an allopolyploid resulting from the interspecific hybridization of two diploid species, the ancestors of *Brassica rapa* and *Brassica oleracea* (Chalhoub *et al*., 2014). The Arabidopsis and *Brassica* lineages diverged before the genome triplication event (Lysak *et al*., 2005). Therefore, an initial expectation would be to find six OSR paralogues for every gene in the *Arabidopsis thaliana* (hereafter referred to as Arabidopsis) genome. However, a comparison of total gene numbers between the two species reveals only a four‐fold difference [101 040 (Chalhoub *et al*., 2014) and 102 422 (He *et al*., 2017) relative to 25 498 (The Arabidopsis Genome Initiative, 2000)], illustrating at a whole‐genome level the extent of gene loss from the OSR genome.

Here we investigate the retention of genes involved in the floral transition. Flowering time is an important agronomic trait for all *Brassica* crops (Raman *et al*., 2013; Guo *et al*., 2014; Nelson *et al*., 2014; Irwin *et al*., 2016), as different growing regions require varieties with different phenologies. Flowering time has been extensively studied in the model species Arabidopsis (Simpson and Dean, 2002; Andrés and Coupland, 2012; Bouché *et al*., 2016), revealing that many flowering time genes are transcription factors that are involved in multiple interactions (Ratcliffe and Riechmann, 2002; Simpson and Dean, 2002). Given the importance of this trait we investigated if orthologues of Arabidopsis flowering time genes have been preferentially retained relative to other genes in the OSR genome. Preferential retention was previously shown for genes involved in the circadian rhythm in paleopolyploid *B. rapa* (Lou *et al*., 2012). Interestingly, the number of OSR orthologues for some Arabidopsis flowering time genes exceeds the expected number of six (Schiessl *et al*., 2014), suggesting that not only have these genes been retained following whole‐genome duplication (WGD), but that additional copies, probably arising from more recent small‐scale duplication events (involving just one or a few genes), have also been retained. Aspects of flowering time control are known to be conserved between Arabidopsis and OSR (Robert *et al*., 1998; Tadege *et al*., 2001; Guo *et al*., 2014). This makes OSR an interesting and agronomically important model for investigating retention and function of flowering time genes following gene multiplication.

Data from a transcriptomic time series (global gene expression in the first true leaf and shoot apex prior to and during the floral transition in OSR) are consistent with preferentially retained flowering time genes being functional. Through comparative gene expression and cluster analysis we demonstrate differences in the expression dynamics of many flowering time gene homologues, suggesting that differential regulation may be important for their retention. As an example, using knowledge of *cis*‐regulatory elements downstream of the Arabidopsis TERMINAL FLOWER 1 (*AtTFL1*) gene, we identify sequence variation that correlates with regulatory differences observed for orthologues of *AtTFL1* in OSR. This case study highlights the importance of distinguishing the expression dynamics of specific homologues for characterizing gene regulation in polyploid crops.

## Results

### OSR expresses a greater number of flowering time gene homologues relative to the whole genome

**Figure 1:**
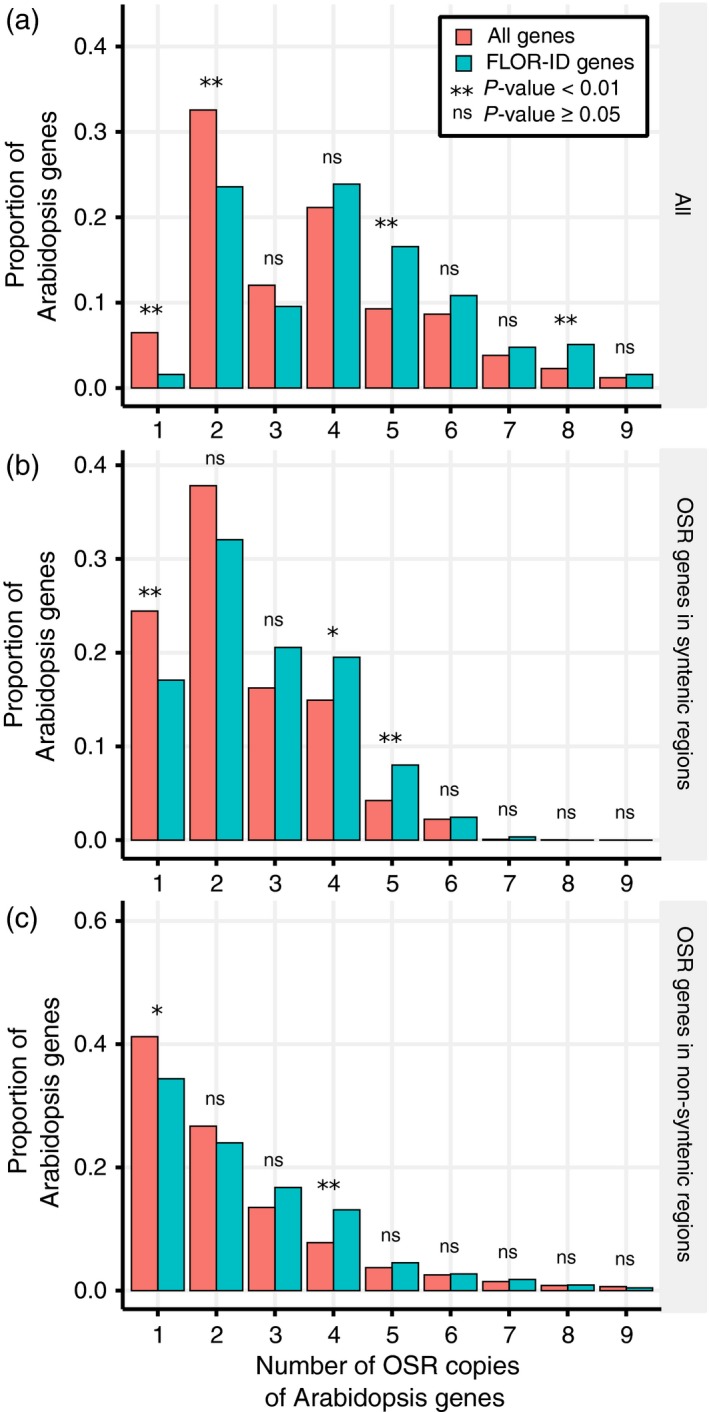
Arabidopsis flowering time genes have been maintained in the oilseed rape (OSR) genome at a higher copy number relative to other Arabidopsis genes. OSR genes were assigned to an Arabidopsis gene by taking the highest scoring BLAST result. The proportions were calculated by counting the number of Arabidopsis genes with a given number of identified OSR copies and dividing by the total number of Arabidopsis genes represented by at least one gene in OSR. The FLOR‐ID distribution is calculated using a subset of 315 Arabidopsis genes annotated as being involved with flower development or flowering time control in the FLOR‐ID database (Bouché *et al*., 2016). *P‐*values were calculated using a two‐sample test for the equality of proportions, performed using the prop.test function in the R statistical programming language (R Core Team, 2017). These plots were generated using all OSR genes with an assigned Arabidopsis gene (a), only OSR genes located in regions of the OSR genome syntenic with regions of the Arabidopsis genome (b), and only OSR genes not located in regions of the OSR genome syntenic with regions of the Arabidopsis genome (c).

To test whether flowering time genes have been preferentially retained in OSR, we compared the numbers of Arabidopsis orthologues in OSR for the entire genome and for the subset of flowering time genes. We found that a statistically significant higher proportion of Arabidopsis flowering time genes have six and eight orthologues in the OSR genome when compared with all Arabidopsis genes, whereas a statistically significant lower proportion of Arabidopsis flowering time genes have one and two orthologues (Figure 1a). To investigate whether the observed retention of flowering time genes is a result of the ancient genome triplication event in the *Brassica* lineage (Chalhoub *et al*., 2014) or due to more recent duplications, we divided orthologues of flowering time gene into those that mapped to syntenic and non‐syntenic locations between OSR and Arabidopsis (Experimental Procedures). By enforcing a 3:1 ratio, these identified syntenic regions likely to have originated from the ancient genome triplication event rather than more recent duplications. Assessing retention of flowering time genes for OSR genes within (Figure 1b) and outside (Figure 1c) these syntenic regions identifies statistically significant similar patterns of retention in both, namely an over‐representation of Arabidopsis genes with high numbers of OSR genes and an underrepresentation of Arabidopsis genes with low numbers of OSR genes. This suggests that both the ancient triplication and more recent small‐scale duplications have contributed to the observed retention of flowering time genes. It should be noted, however, that the assignation of genes to non‐syntenic regions may be due to them being located in regions of the OSR genome which are difficult to assemble.

**Figure 2:**
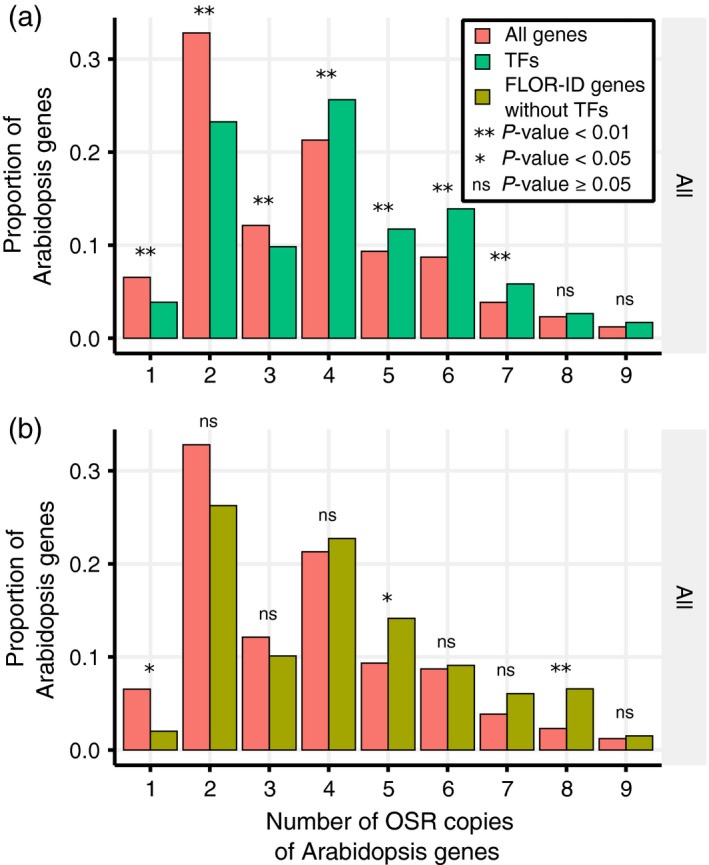
Transcription factors (TFs) have been retained as multiple copies in the oilseed rape (OSR) genome. The proportions of Arabidopsis transcription factors that have a given number of homologues identified in OSR. *P‐*values are computed using the same method as Figure 1 for (a) the subset of Arabidopsis transcription factors, identified using the Plant Transcription Factor Database (version 4.0) (Jin *et al*., 2017) and (b) the subset of floral genes from the FLOR‐ID database (Bouché *et al*., 2016) that are not also in the Plant Transcription Factor Database.

Certain families of genes are predicted, and have been observed, to exhibit a higher incidence of retention following duplication (Maere *et al*., 2005). Transcription factors, for instance, have been predicted by the gene dosage hypothesis (Papp *et al*., 2003; Veitia, 2003, 2004; Veitia *et al*., 2008; Birchler and Veitia, 2012; Veitia and Potier, 2015) to be retained as they often function as protein dimers (Amoutzias *et al*., 2008). Consistent with this, transcription factors in OSR have higher numbers of homologues than average (Figure 2a). As 37% of the Arabidopsis flowering time genes used in this study were transcription factors, we investigated whether this could account for the observed retention of flowering time genes in OSR. However, the exclusion of transcription factors from the flowering time set showed that significant differences in under‐ or over‐representation of flowering time gene copies remained for those present as one, five and eight copies (Figure 2b). Therefore, our findings suggest that retention of transcription factors is only partially responsible for the observed retention of flowering time genes and that other factors are also at play.

**Figure 3:**
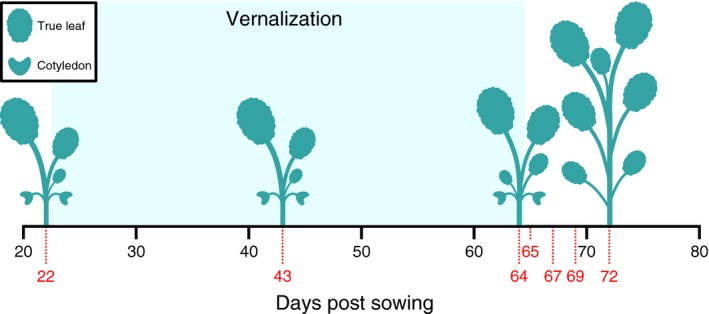
Tissue samples were collected for RNA sequencing at selected points through development. Plants were grown as detailed in the Experimental Procedures. Tissue was sampled on the days indicated with red dotted lines and numbers. The plant silhouettes represent the approximate number of full leaves at the indicated points in development.

However, this counting of genes merely tells us the number of predicted genes in the genome and does not consider whether they are functional. As a step towards addressing this, we asked which of the retained flowering time gene copies are expressed, and therefore potentially functional. We collected gene expression data (from leaf and shoot apex tissue using RNA‐Seq) through the vegetative to reproductive transition from BBCH stage 13 to BBCH stage 51 (Lancashire *et al*., 1991) in a doubled haploid (DH) line derived from the spring OSR variety Westar (Figure 3). We considered a gene to be expressed if its maximum expression level during the developmental time series was equal to or exceeded 2 FPKM, with leaf and shoot apex samples tested separately.

Quantifying expression levels of highly similar genes using RNA‐Seq can potentially be biased by short reads originating from sub‐sequences that are identical between the copies and which could map equally well to any copy. To minimize this effect, the sequencing reads were filtered to include only those reads which have a single location in the genome (Experimental Procedures). Repeating the expression quantification using only these uniquely mapping reads resulted in very similar FPKM values (Figure S4) and confidence interval sizes (Figure S5) relative to the values obtained when all reads were used for quantification. This suggests that the uncertainty in read mapping does not have an appreciable effect on the expression values obtained. Throughout the rest of the study, FPKM values calculated using all reads are reported.

**Figure 4:**
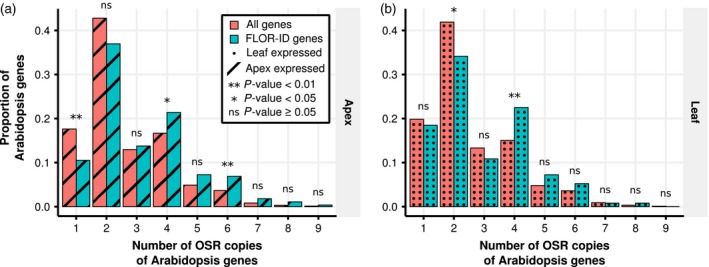
Multiple oilseed rape (OSR) flowering time gene homologues are expressed during the floral transition. The proportions of Arabidopsis genes that have a given number of homologues which are expressed in OSR. OSR genes were considered to be expressed if their maximal expression level within a tissue across the time series was above 2.0 FPKM (fragments per kilobase of transcript per million mapped reads). *P*‐values are computed in the same way as Figure 1. (a) OSR genes expressed in the apex tissue that show sequence conservation to an annotated Arabidopsis gene. (b) OSR genes expressed in the leaf tissue that show sequence conservation to an annotated Arabidopsis gene.

In both shoot apex and leaf (Figure 4a, b), a shift towards the expression of a higher number of flowering time gene homologues, relative to the whole genome, is observed, with a significant over‐representation of FLOR‐ID genes (Bouché *et al*., 2016) with four and six copies. To test if this finding was due to the retention of circadian genes, as has been reported in *B. rapa* (Lou *et al*., 2012), we repeated this analysis after removing circadian genes. We found that the pattern remained with a significant over‐representation of flowering time genes present at five or eight copies (Figure S6). This confirms the observation that flowering time genes, and not just those involved in circadian regulation, have been retained in OSR. Furthermore, the over‐representation of expressed flowering time genes suggests that many of the multiple orthologues of Arabidopsis flowering time genes retained could be functional.

### Differences in spatio‐temporal expression suggest regulatory divergence of flowering time gene homologues in OSR

**Figure 5:**
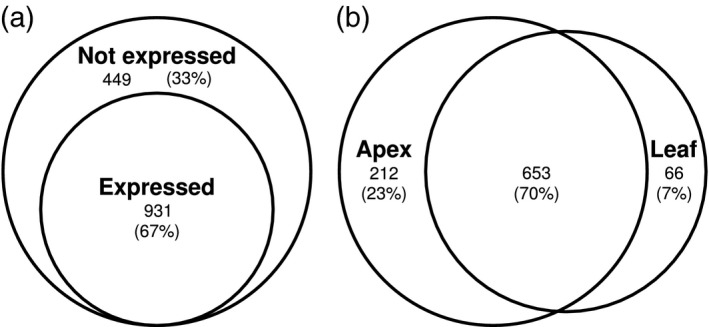
Oilseed rape (OSR) flowering time genes exhibit differential and tissue‐specific expression. (a) Euler diagram showing that 67% of annotated OSR flowering time genes are expressed in either the leaf or the apex during the transcriptomic time series. Genes were considered expressed if their maximal expression level within a tissue across the time series was above 2.0 FPKM (fragments per kilobase of transcript per million mapped reads). (b) Venn diagram showing the extent of tissue‐specific expression of OSR flowering time genes during the transcriptomic time series.

**Figure 6:**
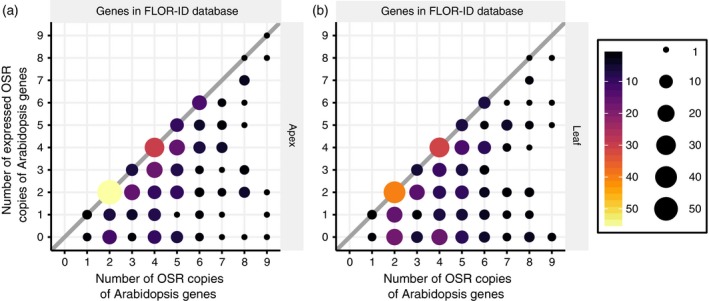
Not all annotated oilseed rape (OSR) orthologues of Arabidopsis flowering time genes are expressed. Expression data from the apex (a) and leaf (b) show that not all OSR copies of Arabidopsis genes were expressed in the developmental transcriptome time series. The size and colour of the circles indicate the number of data points at that position in the graph. The thick diagonal line indicates Arabidopsis genes that have OSR orthologues that are all expressed during the developmental transcriptome. Only OSR genes that show sequence conservation to an annotated Arabidopsis genes present in the FLOR‐ID database (Bouché *et al*., 2016) were used to generate these results. A similar graph generated using all OSR genes that show sequence conservation to an annotated Arabidopsis gene is illustrated in Figure S8.

Having shown that genes involved in the control of flowering time are retained as multiple homologues in the OSR genome, we next used the transcriptomic time series to investigate whether copies of the same flowering time gene show conserved or divergent spatial and temporal expression patterns. Of the 1380 OSR flowering time genes considered in this study, 449 (33%) are not expressed in either the apex or the leaf (Figure 5a). Of the 931 OSR flowering time genes that are expressed, 212 (23%) show apex‐specific expression and 66 (7%) show leaf‐specific expression, with the remaining 653 (70%) being expressed in both tissues (Figure 5b). These findings are significantly different from the pattern of tissue‐specific expression at the genome‐wide level, where 7530 (17%) are apex specific, 4949 (11%) are leaf specific and the remaining 32674 (72%) are expressed in both tissues (*P*‐value = 5.7 × 10^–8^, Pearson's chi‐square test with two degrees of freedom; Figure S7). To relate these findings to OSR orthologues of Arabidopsis flowering time genes, we compared the number of annotated and expressed OSR orthologues for each Arabidopsis flowering time gene. If all OSR orthologues had the same regulation we would expect them all to be expressed in the same tissue (solid diagonal line in Figure 6; see also Figure S8). However, we find that 61% have at least one OSR orthologue that is not expressed in the apex, with this increasing to 69% in the first true leaf (Figures 6 and S8), suggesting that OSR orthologues of flowering time genes have diverged in their regulation.

**Figure 7:**
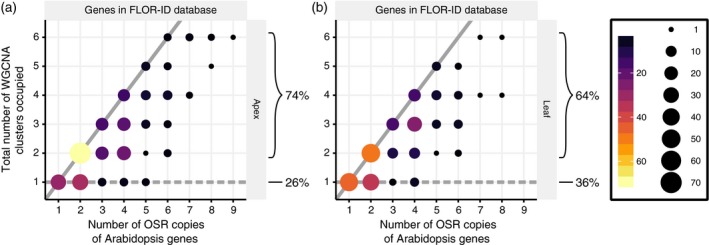
The majority of flowering time gene homologues in oilseed rape (OSR) are assigned to different regulatory modules. Regulatory module assignments for the apex (a) and leaf (b). The size and colour of the circles indicates the number of data points at that position in the graph. The thick lines on each graph represent two potential extremes. The dashed line represents the null hypothesis that all OSR copies of an Arabidopsis gene are assigned to the same weighted gene co‐expression network analysis (WGCNA) cluster. The solid line represents the Arabidopsis genes that have OSR copies that are each assigned to separate WGCNA clusters. The percentages on the graph indicate the percentage of data points that agree, and the percentage that do not agree, with the null hypothesis. Only OSR genes with expression above 2.0 FPKM (fragments per kilobase of transcript per million mapped reads) in at least one time point in the developmental time series and sequence conservation to an annotated Arabidopsis gene were used. A similar graph generated using all OSR genes that show sequence conservation to an annotated Arabidopsi*s* gene is shown in Figure S9.

To further interrogate the extent of regulatory divergence we used WGCNA. This uses normalized expression data to cluster genes together based on their temporal expression profiles rather than expression levels per se. We used these cluster assignments to assess the regulatory control of flowering time gene homologues. Based on the premise of tight co‐regulation of dosage‐sensitive functionally redundant genes (Papp *et al*., 2003), our null hypothesis is that all OSR orthologues of an Arabidopsis flowering time gene will have similar temporal expression patterns, leading to orthologues being in the same regulatory module (dashed lines in Figure 7; see also Figure S9). We found that most OSR flowering time genes (74% in the apex, 64% in the leaf) do not support this hypothesis (Figures 7 and S9) with only 26% in the apex and 36% in the leaf showing correlated expression patterns. Thus, analysis of the overall levels of expression in both leaf and shoot apex, and clustering‐based analysis, reveals both spatial and temporal divergence of expression between retained homologues of flowering time genes in OSR, again suggesting regulatory variation between homologues.

### Self‐organizing maps capture different patterns of regulation for OSR orthologues of the flowering time genes *AtTFL1*,* AtFT* and *AtLFY*

**Figure 8:**
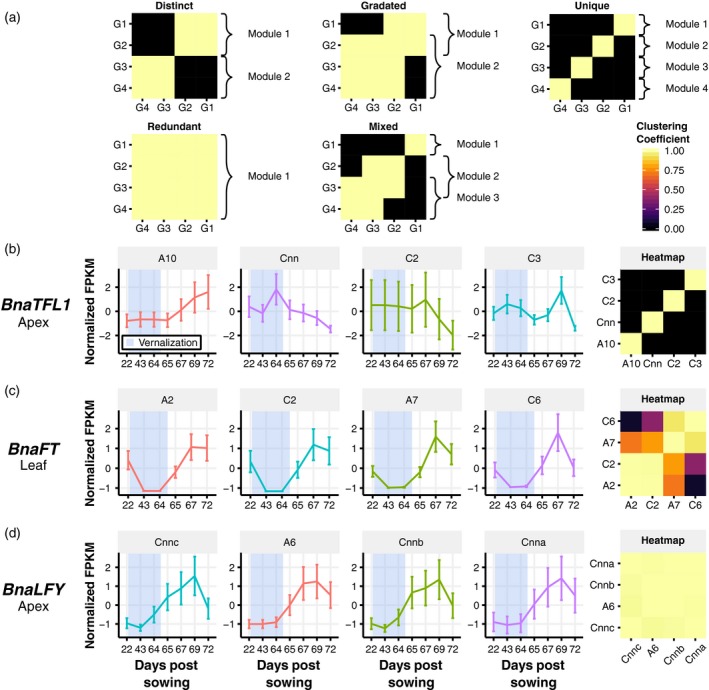
The oilseed rape (OSR) orthologues of *AtTFL1*,* AtFT* and *AtLFY* show different patterns of regulation. (a) Representations of the five patterns of regulatory module assignment detected by the self‐organizing map (SOM)‐based method. High clustering coefficients between two different genes indicates that those genes have similar expression traces. Clustering coefficients between a gene and itself represent how robustly a gene maps to the SOM. A *distinct* pattern indicates multiple regulatory modules being identified, with no gene occupying more than one module. A *gradated* pattern represents multiple regulatory modules being detected, but genes occupy multiple modules. *Redundant* patterns occur when only one regulatory module is detected, and all copies of a gene are assigned to that module. *Unique* patterns are a special case of the *distinct* pattern where each copy of a gene is assigned to a different regulatory module. *Mixed* patterns consist of a mixture of *distinct* and *gradated* patterns, where the gene assignments of some modules overlap while others do not show overlap. When assessing the regulatory module assignment, gene copies that do not robustly map to the SOM are removed. (b)–(d) Expression traces across the developmental time series were normalized to a mean value of 0.0 FPKM (fragments per kilobase of transcript per million mapped reads) and unit variance across the time series. The shading indicates time points during which the plants were grown in cold conditions. Regulatory module assignment heatmaps calculated using the SOM‐based method for the OSR copies of *TFL1*,* FT* and *LFY* are also displayed. Both the expression traces and the clustering coefficients are apex derived for *TFL1* (b) and *LFY* (d) and leaf derived for *FT* (c).

Whilst WGCNA assigns expression profiles to regulatory modules, the similarity between profiles is not quantified and genes that could be assigned to multiple regulatory modules are only assigned to a single module. Furthermore, WGCNA does not account for uncertainty in the RNA‐Seq data in the assignment of regulatory modules. To address these issues, we developed a SOM approach to assess potential differences in regulation between gene homologues (Data S1, Figure S2). Figure 8(a) illustrates the five possible patterns of regulatory module assignment: (i) a *distinct* pattern of multiple regulatory modules with genes assigned to a single module; (ii) a *gradated* pattern of multiple modules where gene membership of individual modules overlap; (iii) a *unique* pattern (a special case of the *distinct* pattern) where each copy of a gene is assigned to a different module; (iv) a *redundant* pattern where all genes are assigned to the same regulatory module; (v) a *mixed* pattern with some modules showing overlap in gene membership and others not. This approach allows us to robustly analyse expression similarity.

Of the 85 pairs of homologues expressed in the apex, 67 (79%) are found in the same regulatory module. In the leaf, 53 of 69 (77%) expressed homologous pairs are found in the same module, with 29 of these pairs being common between the two tissues (File S1). Thus, despite 64% (apex) and 74% (leaf) of flowering time gene homologues exhibiting divergence in their expression dynamics, equivalent genes from the A and C genome (homologous pairs) frequently have similar expression profiles. The percentage of Arabidopsis genes with at least two expressed homologues in the apex (leaf) exhibiting each of the regulatory module assignments are 25% (26%) *distinct*, 9% (6%) *gradated*, 23% (23%) *unique*, 39% (33%) *redundant* and 3% (6%) *mixed* (Figure S2).

To investigate this further we focused on three Arabidopsis flowering time genes, *AtLFY*,* AtFT* and *AtTFL1*, which are known to be central to the regulatory network responsible for the transition to flowering in rapid cycling Arabidopsis (Jaeger *et al*., 2013). Each of these genes has four expressed orthologues in OSR, with *BnaTFL1* and *BnaLFY* being expressed in the apex and *BnaFT* in leaf tissue. A SOM analysis revealed that orthologues of *AtLFY*,* AtFT* and *AtTFL1* in OSR exhibit three different patterns of regulatory module assignment: *redundant*,* gradated* and *unique*, respectively.

The expression dynamics of homologues of *BnaLFY* gave rise to a regulatory module with a *redundant* pattern, with each of the expression profiles in the apex showing low expression initially and an increase after the vernalization period (Figure 8d), analogous to observations of *AtLFY* expression in Arabidopsis (Blazquez *et al*., 1997). Similar expression dynamics of *BnaLFY* homologues is consistent with the gene balance hypothesis (Papp *et al*., 2003; Birchler and Veitia, 2012) and suggests that *AtLFY* displays dosage sensitivity (Okamuro *et al*., 1996; Blazquez *et al*., 1997).

The four *BnaFT* homologues exhibit a *gradated* pattern with two modes of regulation (Figure 8c). The expression of all homologues of *BnaFT* decreases during vernalization and returns to pre‐vernalization levels when the plants are returned to growth in warm, long‐day conditions. The *BnaFT* expression profiles diverge at the final time point (day 72, BBCH stage 51) with the A7 and C6 homologues showing a pronounced decrease in expression between days 67 and 72. The decrease in expression of *BnaFT.A7* is less than that of its homologue, resulting in assignment to both regulatory modules. The *BnaFT* homologues expressed in the leaf therefore exhibit a gradient of regulatory responses, with *BnaFT.A2* and *BnaFT.C2* having divergent expression traces relative to *BnaFT.C6*, but with *BnaFT.A7* showing similarities to all homologues.

The OSR orthologues of *AtTFL1* are an example of *unique* regulatory module assignment with each of the four *BnaTFL1* genes assigned to different modules (Figure 8b). *BnaTFL1.A10* is expressed before and during cold, with an immediate increase in expression when the plants are returned to growth in warm, long‐day conditions. *BnaTFL1.C2* also exhibits stable expression before and during cold, but in contrast to *BnaTFL1.A10* it decreases in expression when the plants are returned to warm, long‐day conditions. *BnaTFL1.C3* exhibits reduced expression levels post‐cold with a transient peak of expression at day 69. The fourth homologue (mapped to the Darmor‐*bzh* C genome and with greatest sequence identity to *BolTFL1.C9* from the EnsemblPlants database; Kersey *et al*., 2016) shows increased expression during cold followed by a steady decrease when plants are returned to warm, long‐day conditions. These four expression profiles are *unique*, as shown in the clustering coefficient heatmap (Figure 8b). Homologues *BnaTFL1.A10* and *BnaTFL1.C3* exhibit expression profiles with the greatest similarity to *AtTFL1* (Ratcliffe *et al*., 1999) with both showing increasing expression during the floral transition.

*AtLFY*,* AtFT* and *AtTFL1* integrate environmental signals to determine the timing of the floral transition (Schultz and Haughn, 1991; Shannon and Meeks‐Wagner, 1991; Huala and Sussex, 1992; Kardailsky *et al*., 1999; Kobayashi *et al*., 1999). That individual orthologues of these genes in OSR show different patterns of regulatory module assignment suggests that the selective pressures acting on them are different, even though they belong to the same regulatory pathway in Arabidopsis. This result mirrors findings in Arabidopsis where it was found that less than half of gene pairs derived from the most recent duplication still retained significantly correlated expression profiles (Bowers *et al*., 2003; Blanc and Wolfe, 2004).

### Patterns of intergenic sequence conservation surrounding *BnaTFL1* genes provide a potential explanation for the observed regulatory divergence

**Figure 9:**
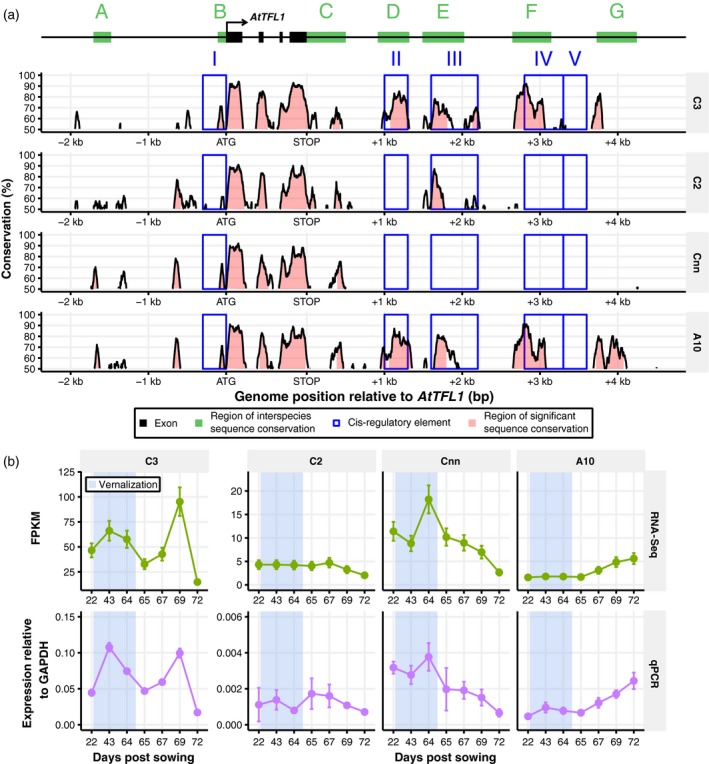
‐ Sequence analysis reveals that *cis*‐regulatory modules identified in Arabidopsis are not present downstream of some copies of *TFL1* in oilseed rape (OSR). (a) The degree of sequence conservation between the OSR copies of *TFL1* and *AtTFL1*. Sequence alignment and conservation calculations were performed using the mVISTA server (Mayor *et al*., 2000; Frazer *et al*., 2004) with a sliding window size of 100 bp. The seven regions of high interspecies sequence conservation (green bars) and the five *cis*‐regulatory regions (blue boxes) identified (Serrano‐Mislata *et al*., 2016) by Serrano‐Mislata *et al*. are shown relative to the *AtTFL1* gene model (black bars). The labelling of these regions follows the same conventions as the previous study. The pink shaded areas under the sequence conservation curves are regions above 70% sequence conservation. Genomic positions upstream and downstream of the *TFL1* gene copies are given relative to the ATG and STOP codon sites, respectively. (b) The unnormalized expression traces for the *BnaTFL1* genes determined through RNA sequencing and reverse transcription quantitative PCR (RT‐qPCR). The expression values calculated for RT‐qPCR are normalized to *GAPDH* with the error estimated from the pooled biological replicates (see Experimental Procedures).

Downstream regulatory sequences of *AtTFL1* in Arabidopsis have been shown to be important for spatio‐temporal control of expression (Serrano‐Mislata *et al*., 2016). We therefore investigated whether similar variation could explain the distinct pattern of regulation displayed by the four *BnaTFL1* orthologues. We analysed sequence conservation between OSR and Arabidopsis in the 5′ and 3′ intergenic regions surrounding *BnaTFL1*, identifying several conserved regions (Figure 9). Focusing on areas previously identified as *AtTFL1 cis*‐regulatory elements in Arabidopsis (Serrano‐Mislata *et al*., 2016), we found variation in the degree of sequence conservation between *BnaTFL1* orthologues (Figure 9a). Sequence conservation within regions II and IV of *BnaTFL1.A10* and *BnaTFL1.C3* suggests that Arabidopsis‐like *cis*‐regulatory elements are present downstream of these genes. Region II was found to be necessary for the upregulation of *AtTFL1* during the floral transition in Arabidopsis (Serrano‐Mislata *et al*., 2016). Consistent with Arabidopsis, these *BnaTFL1* orthologues that increase in expression during the floral transition (*BnaTFL1.A10* and *BnaTFL1.C3*) show high sequence conservation in region II. Conversely, *BnaTFL1.Cnn* and *BnaTFL1.C2*, which are not upregulated during the floral transition, lack sequence conservation in this region. Region IV may also be involved in the observed divergence of the expression trace between *BnaTFL1* homologues, as this region was found to be important for the expression of *AtTFL1* in the inflorescence meristem.

Sequence conservation within region III is below 50% in *BnaTFL1.Cnn*, whilst for the other three homologues it is 81% 87% and 78% for *BnaTFL1.A10*,* BnaTFL1.C2* and *BnaTFL1.C3*, respectively. Interestingly, the range of significant sequence conservation in *BnaTFL1.C2* (154 bases) and *BnaTFL1.A10* (162 bases) is decreased compared with that of *BnaTFL1.C3* (273 bases), potentially suggesting that the *cis*‐regulatory elements in the former two copies are incomplete.

Serrano‐Mislata *et al*. (2016) identified additional regions of sequence conservation across species that were not experimentally implicated in the regulatory control of *AtTFL1* (green shading in Figure 9). We observe sequence divergence in one of these regions, region G. Interestingly it is *BnaTFL.A10* and *BnaTFL1.C3*, which exhibit expression profiles most like that of *AtTFL1*, that show sequence conservation in this region. *BnaTFL1.A10* exhibits high sequence conservation relative to Arabidopsis across this entire region, while *BnaTFL1.C3* shows conservation over about 50% of the region. As with regions II and IV, *BnaTFL1.C2* and *BnaTFL1.Cnn* lack a conserved sequence in region G. We also identified a region of conservation not annotated in the previous analysis of *AtTFL1 cis*‐regulatory elements. This region, situated about 600 bp upstream of the transcription start site of *AtTFL1*, shows about 80% sequence conservation relative to Arabidopsis in *BnaTFL1.A10*,* BnaTFL1.C2* and *BnaTFL1.Cnn*. In *BnaTFL1.C3*, sequence conservation in this region is about 55%.

To validate the observed differences in expression dynamics between the *BnaTFL1* orthologues from RNA‐Seq analysis, we performed copy‐specific RT‐qPCR across the developmental time series (Figure 9b). The RT‐qPCR results show good correspondence with the RNA‐Seq results, confirming our findings. Thus, using sequence conservation we predict the presence/absence of *cis*‐regulatory elements downstream of the *BnaTFL1* genes that may confer similar regulatory control in OSR as in Arabidopsis. *BnaTFL1* orthologues contain different combinations of *cis*‐regulatory elements, which have the potential to underlie the divergent expression traces that they exhibit.

## Discussion

Whole‐genome duplication events are thought to have occurred in most, if not all, angiosperm lineages (Soltis *et al*., 2009) and are well documented in the Brassicaceae (Beilstein *et al*., 2010; Hohmann *et al*., 2015). Whole‐genome triplication (Lysak *et al*., 2005; Beilstein *et al*., 2010; Wang *et al*., 2011) and interspecific hybridization events (Chalhoub *et al*., 2014) have resulted in extensive gene multiplication in *Brassica* species relative to the Arabidopsis lineage. Whole‐genome duplication is considered as a driving force in angiosperm diversification (Jiao *et al*., 2011), introducing genetic redundancy and allowing the evolution of novel gene functions and interactions, leading to neo‐ and subfunctionalization. Whole‐genome duplications are usually followed by a process of ‘diploidization’ (Mandáková *et al*., 2010) that includes genome downsizing (Leitch and Bennett, 2004), chromosome rearrangement and number reduction (Lysak *et al*., 2006), and gene loss (Edger and Pires, 2009). So, whilst many additional gene copies gained from WGD are likely to be lost over time, the analysis of genomic sequences has revealed that a significant number of duplicated genes are nevertheless present in the genomes of many species (Simillion *et al*., 2002; Blanc and Wolfe, 2004; Blomme *et al*., 2006; Freeling, 2009). For instance, in the Arabidopsis lineage around 30–37% of homologous gene duplicates have been retained (Paterson, 2005; Bomblies and Madlung, 2014). Based on such observations, modelling studies have determined conditions under which duplicated genes can become evolutionarily stable (Nowak *et al*., 1997; Veitia, 2003). These ideas have given rise to the gene balance hypothesis, which states that dosage‐sensitive genes are preferentially retained in the genome after WGD but tend to be lost after local duplication events (Papp *et al*., 2003; Birchler and Veitia, 2012). Kinases, transcription factors and proteins that form part of a complex fall into this category of dosage‐sensitive genes. We might therefore expect that highly networked genes such as those that regulate flowering time (Simpson and Dean, 2002; Higgins *et al*., 2010; Bouché *et al*., 2016) have been preferentially retained in the genome.

This study reveals that flowering time genes are more likely to be retained in the OSR genome relative to other genes in the genome (Figure 1). This retention is partially due to a large percentage of flowering time genes being transcription factors (Figure 2). However, the retention of flowering time genes is still observed when transcription factors are removed, suggesting that other factors are influencing the retention of flowering time gene orthologues. An investigation of the previously reported retention of circadian clock genes in *B. rapa* (Lou *et al*., 2012) proved inconclusive in explaining this observation (Figure S6). One potential explanation is that the time since the various genome multiplication events that have occurred in OSR has not been sufficient for loss of redundant gene copies to occur. Redundant gene copies are predicted to remain in the genome as a function of the genetic mutation rates (Nowak *et al*., 1997). If mutation rates are similar, then redundant copies of the gene may be retained in the population for over 10^7^ generations, a length of time which is consistent with the predicted genome triplication in the Brassica lineage 5–28 million years ago (Lysak *et al*., 2005; Beilstein *et al*., 2010; Wang *et al*., 2011). However, there is no a priori reason to expect this effect to preferentially retain flowering time gene orthologues over other OSR homologues. Interestingly, if we restrict the analysis to flowering time genes that mapped to syntenic and non‐syntenic locations between OSR and Arabidopsis, we find similar, statistically significant, patterns of retention in both. This suggests that not only ancient triplication but also more recent small‐scale duplications have contributed to the observed retention of flowering time genes. The retained flowering time genes may of course have diverged in function, and have been retained by selection, artificial or otherwise. Subfunctionalization of gene regulation is predicted to occur on the scale of 4–12.5 million years, under the duplication–degeneration–complementation model (Force *et al*., 1999). These time scales are also consistent with the timings of the gene duplication events during the evolutionary history of OSR. As different gene retention processes are predicted to display differing patterns of selection (Conant and Wolfe, 2008), an interesting avenue for future studies would be to look for signs of selection for the OSR flowering time genes using comparative studies of the OSR genome.

In addition to gene retention, we investigated the expression profiles of OSR genes prior to and during the floral transition. We compared the spatio‐temporal expression dynamics across development to infer whether orthologues of Arabidopsis flowering time genes retain similar patterns of regulation. Whilst our analysis reveals that a significant proportion of duplicated genes in OSR have divergent regulation (Figures 6, 7 and S2b, c), it also shows that the more recently combined homologues are frequently found in the same regulatory module (79% in the apex and 77% in the leaf). An analysis of 2000 pairs of paralogous genes in *Gossypium raimondii*, resulting from a five‐ to six‐fold ploidy increase about 60 million years ago, revealed that more than 92% of gene pairs exhibited expression divergence (Renny‐Byfield *et al*., 2014). Most of these gene pairs show complementary expression patterns in different tissues, consistent with the idea of responsive backup circuits (Kafri *et al*., 2005, 2006). It is therefore tempting to speculate that regulation of homologues in OSR is still in flux with near‐complete divergence being a likely consequence of ‘diploidization’ across much longer timeframes. This hypothesis is supported by the finding that in recently synthesized allotetraploid cotton most homologues display similar expression patterns across multiple tissue types (Adams *et al*., 2003) while in allotetraploid upland cotton (*G. hirsutem*; which arose 1–2 million years ago) 24% of homologues show diverged expression patterns. Recent genomic studies also support the idea that the OSR genome is in flux (Bayer *et al*., 2017; He *et al*., 2017), potentially in response to artificial selection for agronomically important traits.

Gene expression can be controlled through a range of mechanisms. This study highlights the potential role that *cis*‐regulatory elements may play in gene regulation. Expression differences in *AtTFL1* orthologues in OSR correlate with the presence and absence of sequence conservation within regions downstream of the gene. Serrano‐Mislata *et al*. (2016) identified these regions as *cis*‐regulatory elements and dissected their roles in the spatio‐temporal regulation of *AtTFL1* (Serrano‐Mislata *et al*., 2016). The expression dynamics of *AtTFL1* exhibited by Arabidopsis mutants lacking the identified *cis*‐regulatory elements show striking similarities to those of *BnaTFL1* orthologues lacking sequence similarity to the elements. This suggests conserved function of *cis*‐regulatory elements between Arabidopsis and OSR, and highlights that such variation can potentially drive the regulatory divergence of gene homologues. Although the patterns of sequence conservation downstream of *AtTFL1* (Serrano‐Mislata *et al*., 2016) are retained in OSR orthologues (Figure 9), we have not demonstrated that the changes in these *cis*‐regulatory elements are causative. The differences in region II correlate with the upregulation of *BnaTFL1* at the floral transition. This region is not conserved in *BnaTFL1.Cnn*, which also lacks high levels of sequence conservation in region III. The sequence in region III is associated with the expression of *AtTFL1* in Arabidopsis lateral meristems (Serrano‐Mislata *et al*., 2016) and thus predicts that *BnaTFL1.Cnn* is not expressed in this tissue. This could be tested by sampling the lateral meristems during development and determining the relative expression levels of the *BnaTFL1* genes in this tissue using RT‐qPCR. In addition, the novel region of sequence conservation identified upstream of *AtTFL1* in this study would require similar experimental approaches to those employed by Serrano‐Mislata *et al*. (2016) to verify or discount the region as a true *cis*‐regulatory element.

We have shown that many OSR orthologues of Arabidopsis flowering time genes differ in their spatio‐temporal expression across the floral transition. Without biochemical data on the proteins encoded by the genes we are not able to distinguish whether homologues with different expression patterns have maintained their original molecular functions (redundant), specialized such that the initial function is split between gene duplicates (subfunctionalization) or developed a novel function (neofunctionalization).

The presence of multiple gene homologues within crop species complicates the direct transfer of insights derived from model species like Arabidopsis to polyploid crops. Knowledge of the regulatory networks that are active in crops will support future breeding efforts by allowing more targeted, homologue‐specific crop improvement strategies. Detailed understanding of the function of specific copies of genes, their regulation and importantly how this functionality is combined to determine crop plasticity will be key for targeted approaches for crop improvement.

## Experimental Procedures

### Plant growth and sample preparation

*Brassica napus* cv. Westar plants were sown on 7 May 2014 in cereals mix (40% medium grade peat, 40% sterilized soil, 20% horticultural grit, 1.3 kg m^–3^ PG mix 14‐16‐18 +  Te base fertilizer, 1 kg m^–3^ Osmocote Mini 16‐8‐11 2 mg + Te 0.02% B, wetting agent, 3 kg m^–3^ maglime, 300 g m^–3^ Exemptor). Plants were grown in unlit glasshouses in Norwich, UK, with glasshouse temperatures set at 18°C during the day and 15°C at night (approximately 16‐h days). On day 22, plants were transferred to a 5°C, short‐day (8 h) controlled environment room. Although Westar is classed as a spring cultivar of OSR it may still show a mild response to the vernalization period (Murphy and Scarth, 1994). After 6 weeks at 5°C, plants were transferred back to unlit glasshouses (approximately 16‐h days) and grown until the plants flowered.

The first true leaf and shoot apex of each plant were sampled at 22, 43, 64, 65, 67, 69 and 72 days after sowing (between BBCH stages 13 and 51; Table S1 in the online Supporting Information). The first true leaves were cut and immediately frozen in liquid nitrogen. The growing shoot apices were dissected using a razor blade on a tile chilled in dry ice before transfer to liquid nitrogen.

Samples were pooled and ground in preparation for RNA extraction. For apex tissue, about 0.1 g of apices was ground as a pool. At early time points, as the apices were smaller, this mass of tissue equated to approximately 20 plant apices, while at later time points approximately 10 apices were pooled. For leaf samples, between 6 and 10 leaf samples from separate plants were pooled and ground. RNA extraction and DNase treatment were performed following the method provided with the E.Z.N.A^®^ Plant RNA Kit (Omega Bio‐tek Inc., http://omegabiotek.com/store/).

Library preparation and RNA sequencing were carried out by the Earlham Institute, Norwich, UK (http://www.earlham.ac.uk/). RNA sequencing (RNA‐Seq) was performed on RNA samples from six time points for leaf tissue and seven time points for apex tissue. An Illumina HiSeq2500 was used to generate 100‐bp single‐end reads with an average of 67 million reads per sample (Table S4). Further RNA samples for five time points in both the leaf and apex were sequenced at a lower average coverage of 33 million reads per sample. Table S1 summarizes the sampling scheme and indicates the time points for which a second pool of samples was sequenced.

### Gene model prediction and read alignment

We used the Darmor‐*bzh* reference genome sequence (Chalhoub *et al*., 2014) combined with our sequencing data to obtain predictions for gene model splice junctions. The gene model prediction software AUGUSTUS (Stanke *et al*., 2008) (version 3.2.2) was used to determine gene models for the Darmor‐*bzh* reference genome. TopHat (version 2.0.13)‐aligned (Kim *et al*., 2013) RNA‐Seq reads from across the entire time series were combined and filtered using the filterBam tool provided with AUGUSTUS. AUGUSTUS used the filtered reads to help estimate intron locations. Arabidopsis‐derived parameters provided with the AUGUSTUS software were used to predict OSR gene models in the Darmor‐*bzh* genome, with default parameters used otherwise.

The RNA‐Seq reads were aligned and expression levels quantified using the Tuxedo suite of software following the published workflow (Trapnell *et al*., 2012). TopHat (version 2.0.13) (Kim *et al*., 2013) with the b2‐very‐sensitive, transcriptome‐only and prefilter‐multihits parameter sets was used to align reads to the Darmor‐*bzh* reference sequence, using the AUGUSTUS‐derived gene models to determine the location of gene models. Cufflinks (version 2.2.1) (Trapnell *et al*., 2013) was used to quantify the expression levels of OSR genes. Data normalization using cuffnorm was performed separately for leaf and apex tissue samples. Apart from the named parameters, default values were used.

### Filtering reads to obtain reads that map to a unique location in the genome

The TopHat‐aligned reads were filtered with SAMtools (Li *et al*., 2009) to obtain reads that map to a unique location in the genome. The aligned reads were filtered by mapping quality (MAPQ) values, with only reads having a MAPQ value of 42 being used. This is the maximum score assigned to reads by TopHat, with reads that contain many mismatches with the reference sequence, or that map equally well to multiple locations in the genome, being assigned lower MAPQ values.

### Identification of sequence similarity between OSR and Arabidopsis gene models

The BLAST algorithm, using the blastn binary (version 2.2.30+) provided by NCBI (Camacho *et al*., 2009) was used to identify sequence similarity between the AUGUSTUS‐derived (Stanke *et al*., 2008) gene models and the published Arabidopsis gene models downloaded from TAIR (version 10) (Berardini *et al*., 2015). The blastn algorithm was run using default parameters, with an e‐value threshold of 10^−50^ used to identify sequence similarity between the AUGUSTUS‐derived OSR gene models and published Arabidopsis ones. For the analysis conducted in this study, only the highest scoring blastn hit was used to identify OSR copies of Arabidopsis genes.

### Homologue pair identification

The method outlined by Chalhoub *et al*. (2014) was used to identify pairs of homologues between the A and C genomes (Chalhoub *et al*., 2014). The Darmor‐*bzh* reference genome was divided into A and C genomes, removing the reference pseudo‐chromosomes which consist of sequence that is unassigned to a specific chromosome. The separated genomes were uploaded to the CoGe portal (Lyons and Freeling, 2008) and the SynMap tool (Lyons *et al*., 2008) was used to identify regions of syntenic genes between the two genomes. Chains of syntenic genes were identified using DAGchainer (Haas *et al*., 2004), allowing a maximum gene distance of 20 between two matches and with a minimum number of four aligned pairs constituting a syntenic block. A 1:1 synteny screen was performed using the QUOTA‐ALIGN (Tang *et al*., 2011) procedure. The synteny screen is necessary to distinguish homologous regions of the genome and paralogous regions which are the result of genome multiplication events which occurred prior to the interspecies hybridization event in the evolutionary history of OSR. Once syntenic genes had been identified using SynMap, a reciprocal sequence similarity filter was applied using the BLAST algorithm. The blastn algorithm was used with default parameters and a 10^−50^ e‐value threshold to assess sequence similarity, and only homologous pairs which were reciprocal best hits in this analysis were considered. This resulted in 14 427 homologous pairs distributed across the entire OSR genome (Figure S1, Table S2, File S1).

### Identifying syntenic regions between the Arabidopsis and OSR genomes

The SynMap tool (Lyons *et al*., 2008) on the CoGe portal (Lyons and Freeling, 2008) was used to identify regions of the Arabidopsis genome (Berardini *et al*., 2015) (version 10) which were syntenic with the OSR Darmor‐*bzh* reference genome (Chalhoub *et al*., 2014). The same parameters as those used to identify homologous pairs of OSR genes were used, except that the QUOTA‐ALIGN (Tang *et al*., 2011) synteny screen was conducted with a 3:1 ratio to reflect the a priori knowledge of genome triplication within the *Brassica* lineage relative to the Arabidopsis lineage (Lysak *et al*., 2005; Beilstein *et al*., 2010)

### Weighted gene co‐expression network analysis

The weighted gene co‐expression network analysis (WGCNA) was carried out using the WGCNA library (version 1.51) (Langfelder and Horvath, 2008) available for the R statistical programming language (version 3.2.2) (R Core Team, 2017). Due to the size of the dataset, WGCNA was performed on clustered data. The expression data were first filtered and normalized for each tissue separately. Any genes with a maximum FPKM (fragments per kilobase of transcript per million mapped reads) value across the time series of less than 2.0 were removed. For the remaining genes, the expression across time was normalized to have a mean of 0.0 and a variance of 1.0. Using the normalized expression values, hierarchical clustering was conducted separately on the leaf and apex data using Euclidean distances between expression traces and a complete agglomeration method. The hierarchical tree was cut into *H* numbers of clusters and the ratio $\frac{\sum_{c=1}^{H}N_{C}{\left({\overset{¯}{x}}_{C}-\overset{¯}{x}\right)}^{2}}{\sum_{g=1}^{N}{\left(x_{g}-\overset{¯}{x}\right)}^{2}}$ was calculated for each tree cut height, where *N* is the total number of genes, *N*_*c*_ is the total number of genes assigned to cluster *c*,* x*_*g*_ is the expression vector for gene *g*, ${\overset{¯}{x}}_{C}$ is the mean expression vector for genes assigned to cluster *c* and $\overset{¯}{x}$ is the global mean of all expression vectors. The expression vectors are defined as $x_{g}=({\hat{\mathrm{FPKM}}}_{g,22},{\hat{\mathrm{FPKM}}}_{g,43},\cdot ,{\hat{\mathrm{FPKM}}}_{g,72}$ where ${\hat{\mathrm{FPKM}}}_{g,t}$ represents the normalized FPKM level of gene *g* at time point *t*, with all time points included in the vector. A ratio of about 0.98 was chosen as a good balance between the number of clusters and how well the clusters represented the expression data. This ratio corresponded to 2683 clusters for leaf tissue and 6692 clusters for apex tissue.

A WGCNA (Langfelder and Horvath, 2008) was carried out using the mean expression vectors for the 6692 apex clusters and the 2683 leaf clusters. Based on the assumption of a scale‐free network structure, a soft threshold of 30 was used for both the apex and leaf samples. A minimum regulatory module size of 30 was used and modules with similar eigengene values were merged to give the final regulatory modules used for regulatory module assignment.

### Self‐organizing maps and the identification of regulatory modules

Self‐organizing maps (SOMs) were generated using the Kohonen library (Wehrens and Buydens, 2007) available for the R statistical programming language (R Core Team, 2017). As with the WGCNA analysis, the data were filtered and normalized prior to carrying out the SOM analysis (Data S1). The number of nodes used in the SOM was chosen based on the ratio $\frac{\sum_{c=1}^{S}N_{C}{\left(x_{C}-\overset{¯}{x}\right)}^{2}}{\sum_{g=1}^{N}{\left(x_{g}-\overset{¯}{x}\right)}^{2}}$ where *N* is the total number of genes, *S* is the total number of SOM nodes, *N*_*c*_ is the total number of genes assigned to SOM node *c*,* x*_*g*_ is the expression vector for gene *g*,* x*_*c*_ is the expression vector for SOM node *c* and $\overset{¯}{x}$ is the global mean of all expression vectors. A value of *S* was chosen such that the above ratio was about 0.85 for both tissues. To adequately capture the variation present in the data, the dimensions of the SOM were set as the ratio between the first two principal component eigenvalues of the data, as has been done previously (Vesanto *et al*., 2000).

To assign probabilities of genes clustering to the same SOM cluster, a resampling procedure was employed (Data S1, Figure S2a). Expression values were sampled assuming a Gaussian noise model, using the expression value as the mean of the distribution and the expression value uncertainty calculated by Cufflinks as the distribution variance. The sampled expression values for each gene, within each tissue, were normalized to a mean expression of 0.0 with a variance of 1.0 across the time series and assigned to a SOM cluster based on a minimal Euclidean distance. This sampling loop was repeated 500 times, and the SOM clusters to which the genes of interest mapped were recorded. From this process, an empirical probability of mapping to each SOM cluster was calculated for each gene of interest. The probability of two genes mapping to the same SOM cluster was then calculated as $\sum_{C=1}^{S}\frac{n_{g1,c},n_{g2,c}}{250000}$ where *S* is the total number of SOM clusters and *n*_*gi,c*_ is the number of times gene *g*_*i*_ mapped to SOM cluster *c*. As the SOM training process begins from a random starting point, some SOMs were found to better discriminate between the expression traces of some pairs of genes than other SOMs. To overcome this, the probability of two genes of interest mapping to the same SOM cluster was calculated for 100 different SOMs. This probability was averaged to give the average probability of two genes of interest mapping to the same SOM cluster.

The probability of mapping to the same cluster can also be calculated for a single gene of interest by calculating $\sum_{c=1}^{S}{\left(\frac{n_{g1,c}}{500}\right)}^{2}$. This value is a measure of how consistently a gene maps to the same SOM cluster, giving an indication of the uncertainty in the expression values calculated for that gene. Plotting a distribution of these self‐clustering probabilities (Figure S3) reveals a bimodal distribution with maxima at about 0.05 and 1.0. To aid with visualizing the average probabilities of two genes mapping to the same SOM cluster, because of this bimodality, a soft threshold based on a cumulative Gaussian density function was applied. The resulting value is referred to in the main text as a clustering coefficient. Clustering coefficients were calculated as $\frac{1}{2}\left[1+\mathrm{erf}\left(\frac{\mu_{p_{g_{1},g_{2}}}-\theta }{\sigma_{p_{g_{1},g_{2}}}\sqrt{2}}\right)\right]$ where erf is the error function, $\mu_{p_{g_{1},g_{2}}}$ is the average probability of genes *g*_1_ and *g*_2_ mapping to the same cluster, $\sigma_{p_{g_{1},g_{2}}}$ is the standard deviation of the probabilities calculated from the 100 different SOMs used in the sampling procedure and θ is the tissue‐specific threshold. A threshold of 0.053 (apex) or 0.056 (leaf) was used. This threshold was calculated by taking the self‐clustering probability that corresponded to the maximum of the density curve (Figure S3) for each SOM and averaging them.

Clustering coefficients were subjected to a binary filter, such that coefficients above 0.5 were set to 1 and those below set to 0. Regulatory modules were defined as groups of genes where the binary clustering coefficients between all genes were 1.

### Sequence conservation analysis of orthologues of *AtTFL1* in OSR

The sequence upstream and downstream of the *AtTFL1* gene was extracted from the AtGDB TAIR9/10 v171 Arabidopsis genome assembly located on PlantGDB (Duvick *et al*., 2008) and from the Darmor‐*bzh* reference genome sequence (Chalhoub *et al*., 2014). Regions of conserved sequence were identified using mVISTA from the VISTA suite of tools (Mayor *et al*., 2000; Frazer *et al*., 2004). The alignment algorithm used was AVID (Bray *et al*., 2003), which performed global pair‐wise alignments for all sequences. Percentage sequence conservation was calculated using a 100‐bp sliding window.

### Quantitative PCR of *BnaTFL1* homologues

Reverse transcription quantitative PCR (RT‐qPCR) was carried out on copies of *TFL1* using custom‐designed primers (Table S3). The SuperScript^®^ III First‐Strand Synthesis System (Thermo Fisher Scientific Inc., https://www.thermofisher.com/) was used to generate cDNA, with 2 μg of RNA used as input. The RNA was extracted as described above. Each RT‐qPCR reaction consisted of 5 μl of LightCycler^®^ 480 SYBR Green I Master (Roche Molecular Systems Inc., https://molecular.roche.com/), 4 μl of cDNA, 0.125 μl of the forward and reverse primers at a concentration of 10 μm and 0.75 μl of water. Quantification was performed on a LightCycler^®^ 480 (Roche Molecular Systems Inc.). The RT‐qPCR cycle consisted of a 95°C denaturation step for 5 min followed by 50 quantification cycles. Each cycle consisted of 15 sec at 95°C, 20 sec at 58°C and 30 sec at 72°C. Fluorescence was quantified at 75°C as the temperature was ramping from 72°C to 95°C. Expression values for *BnaTFL1* genes were calculated relative to the expression of glyceraldehyde 3‐phosphate dehydrogenase (GAPDH).

### Data availability

All sequencing reads collected as part of this study have been made available in the NCBI Sequence Read Archive under BioProject number PRJNA398789.

## Author contributions

JI and RM conceived the project. MJ, NP, RW, MT, JI and RM designed the experiments that were carried out by RW with support from NP, MJ and JI. The sequence analysis was carried out by MJ with help from MT. MJ performed all transcriptomic time‐series analyses and produced all the figures. MJ drafted the manuscript which was planned by MJ, NP, JI and RM. All authors contributed to writing the manuscript.

## Conflict of Interests

The authors declare that they have no conflicts of interest.

## Supporting information


**Figure S1.** Locations of identified homologue pairs in the oilseed rape genome.
**Figure S2.** Self‐organizing map‐based assessment of expression trace divergence uncovers widespread regulatory differences and subtle patterns of divergence.
**Figure S3.** A bimodal distribution of self‐clustering probabilities necessitates the use of a threshold to visualize the probabilities.
**Figure S4.** Reads aligning equally well to multiple locations in the genome have little effect on the estimated gene expression levels.
**Figure S5.** Reads aligning equally well to multiple locations in the genome have little effect on the estimated FPKM (fragments per kilobase of transcript per million mapped reads) confidence interval sizes.
**Figure S6.** The observed retention of flowering time genes is not explained by genes associated with the circadian rhythm alone.
**Figure S7.** Euler and Venn diagrams showing the percentage of expressed genes and the percentage of genes expressed in the apex and leaf samples.
**Figure S8.** Not all annotated oilseed rape copies of Arabidopsis genes are expressed.
**Figure S9.** Many gene copies are assigned to different regulatory modules in oilseed rape.

**Table S1.** Sampling and sequencing scheme for the developmental time series.
**Table S2.** Number of genes expressed two‐fold higher than their homologue for all flowering time gene homologue pairs.
**Table S3.** Quantitative PCR primer sequences.
**Table S4.** Sequencing statistics for the two sequencing runs carried out to generate the developmental transcriptome.

**Data S1.** Supplementary results

**File S1.** Locations of homologous pairs and the tissues in which they are expressed
