# Supplementary material for: Spatio‐temporal expression dynamics differ between homologues of flowering time genes in the allopolyploid Brassica napus
Source: Plant J. 2018 Aug 24;96(1):103–18. doi: 10.1111/tpj.14020 (PMC6175450; doi:10.1111/tpj.14020)
Supplement: Supplementary file 1 — Figure S1. Locations of identified homologue pairs in the oilseed rape genome. Figure S2. Self‐organizing map‐based assessment of expression trace divergence uncovers widespread regulatory differences and subtle patterns of divergence. Figure S3. A bimodal distribution of self‐clustering probabilities necessitates the use of a threshold to visualize the probabilities. Figure S4. Reads aligning equally well to multiple locations in the genome have little effect on the estimated gene expression levels. Figure S5. Reads aligning equally well to multiple locations in the genome have little effect on the estimated FPKM (fragments per kilobase of transcript per million mapped reads) confidence interval sizes. Figure S6. The observed retention of flowering time genes is not explained by genes associated with the circadian rhythm alone. Figure S7. Euler and Venn diagrams showing the percentage of expressed genes and the percentage of genes expressed in the apex and leaf samples. Figure S8. Not all annotated oilseed rape copies of Arabidopsis genes are expressed. Figure S9. Many gene copies are assigned to different regulatory modules in oilseed rape. [file TPJ-96-103-s001.docx]

# *
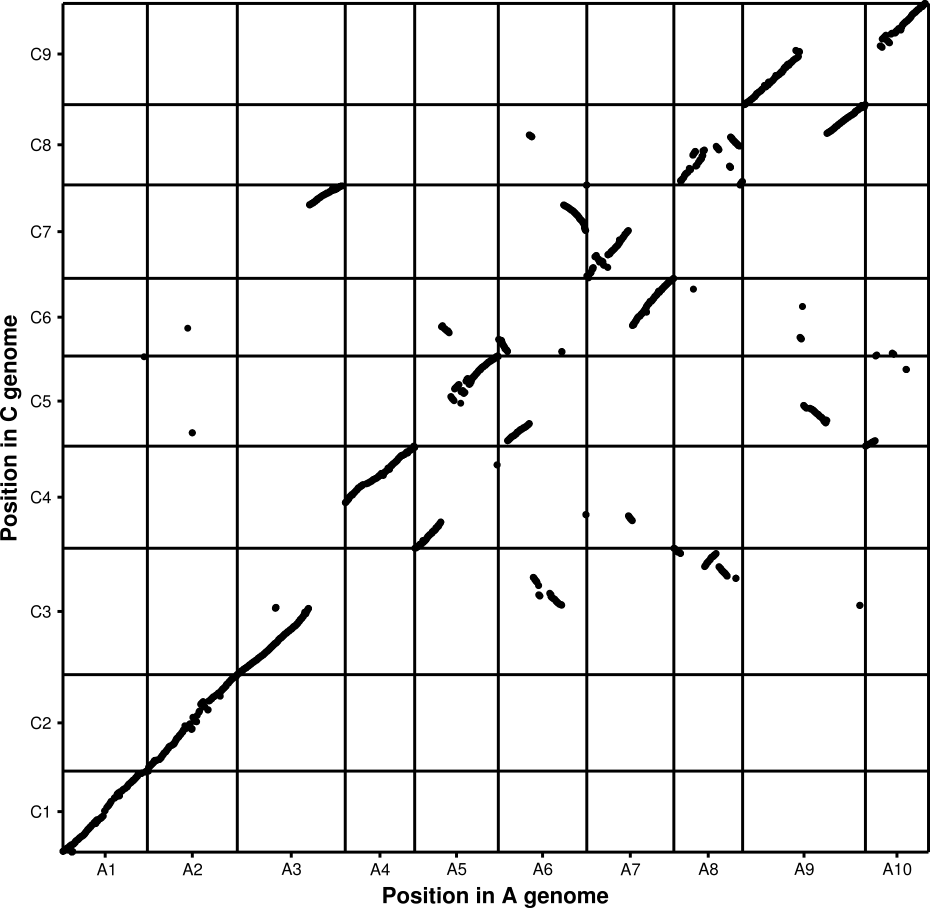
*

## Supplementary figure 1 – Locations of identified homoeologues pairs in the OSR genome.

##
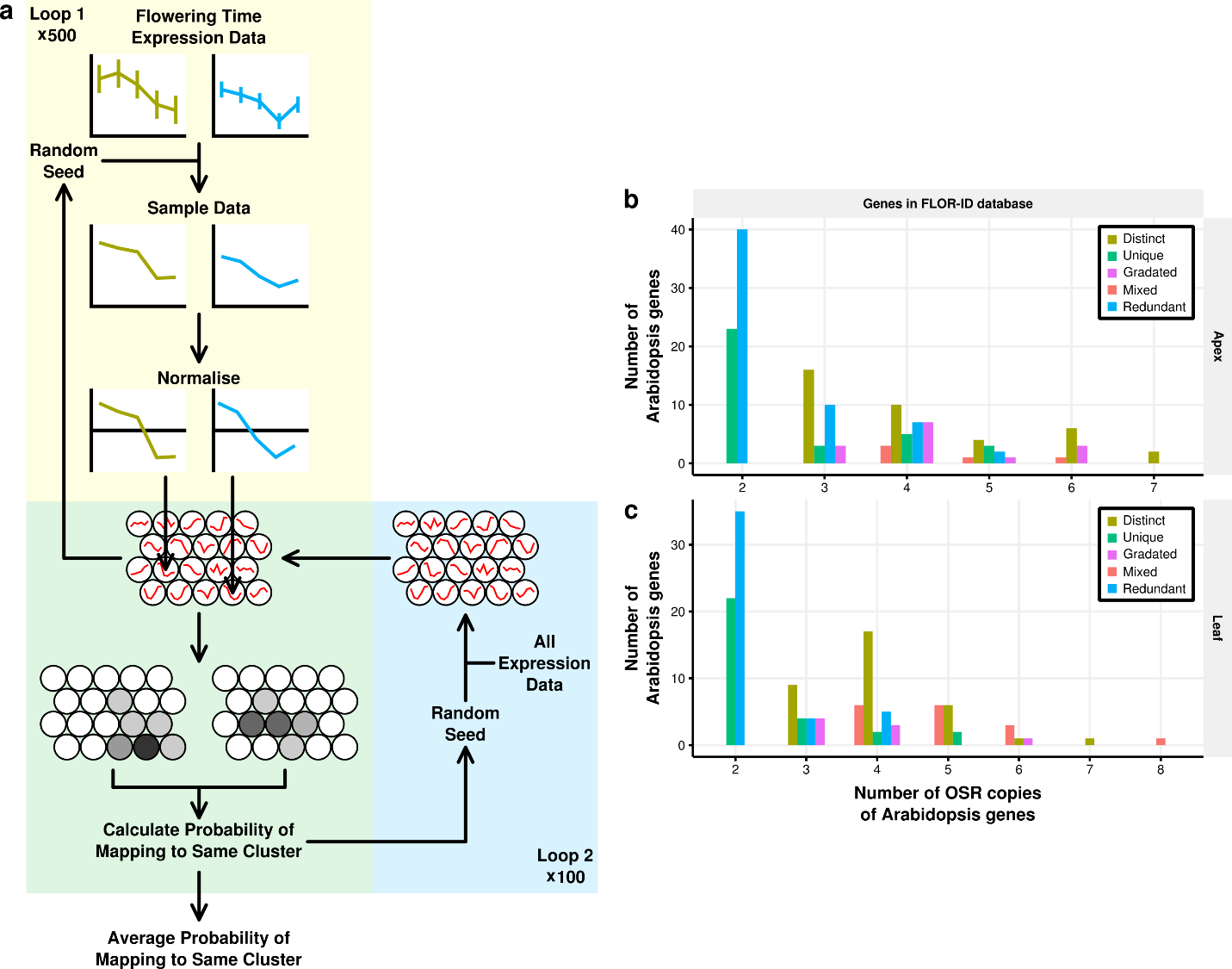
Supplementary figure 2 – Self-organising map (SOM) based assessment of expression trace divergence uncovers widespread regulatory differences and subtle patterns of divergence.

##
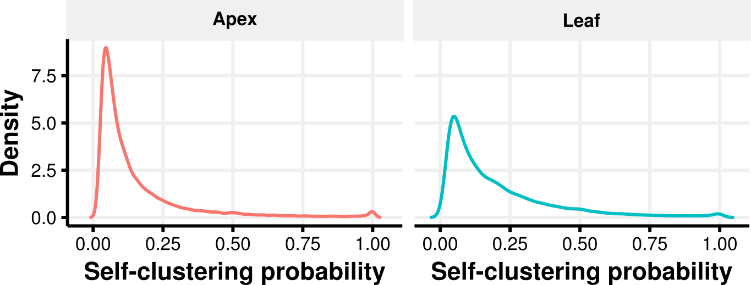
Supplementary figure 3 – A bimodal distribution of self-clustering probabilities necessitates the use of a threshold to visualise the probabilities

##
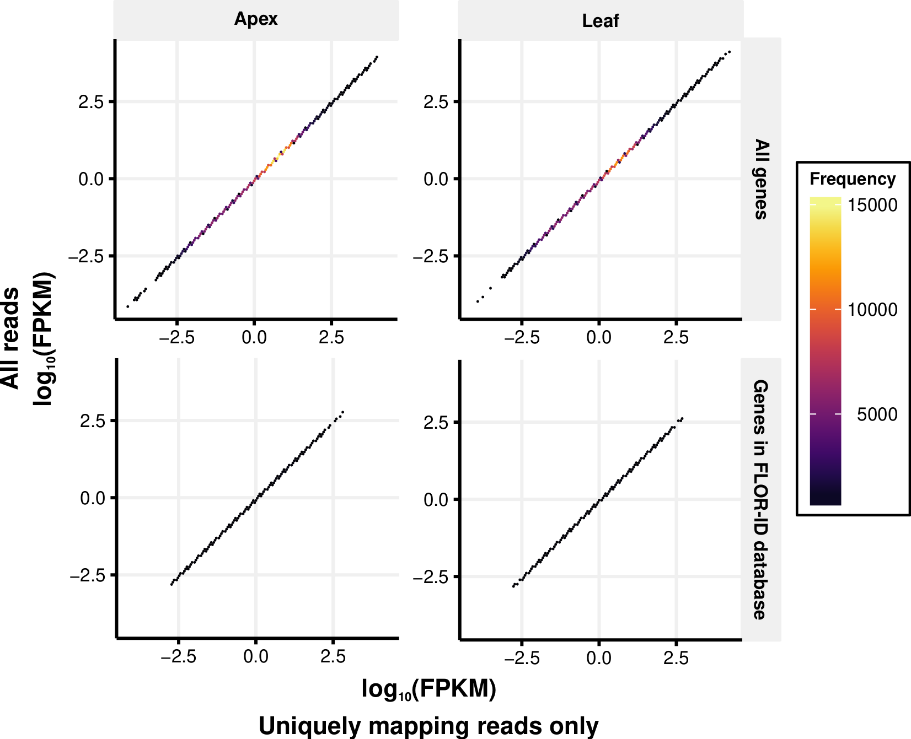
S**upplementary figure 4 –** Reads aligning equally well to multiple locations in the genome have little effect on the estimated gene expression levels.

##
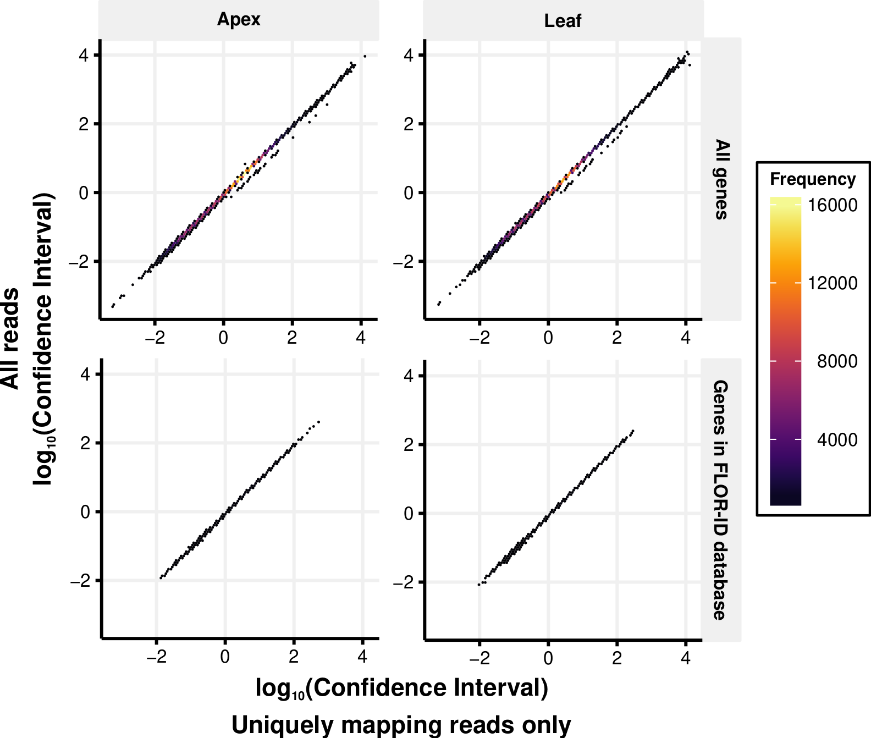
Supplementary figure 5 – Reads aligning equally well to multiple locations in the genome have little effect on the estimated FPKM confidence interval sizes.

##
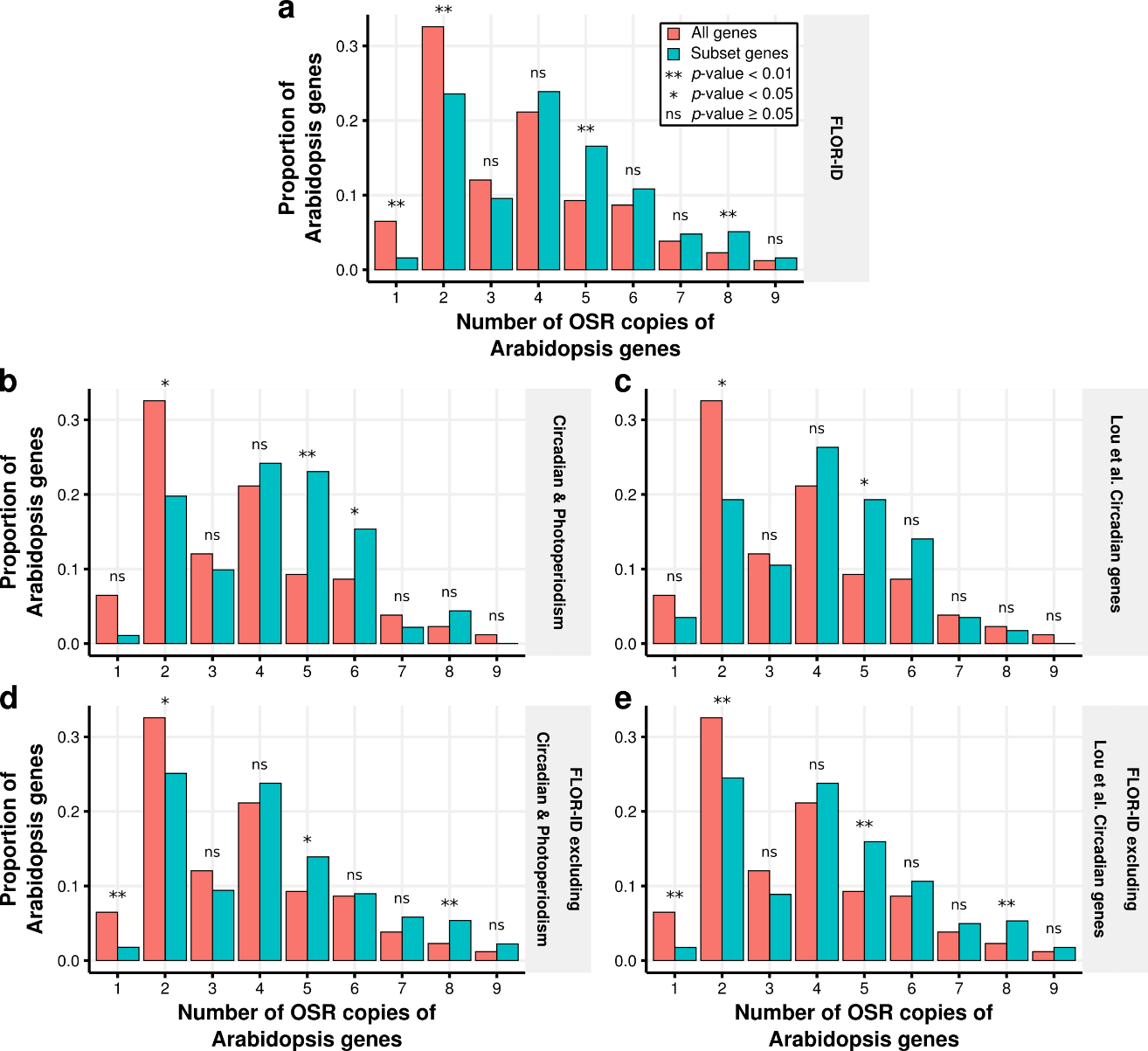
Supplementary figure 6 – The observed retention of flowering time genes is not explained by genes associated with the circadian rhythm alone.


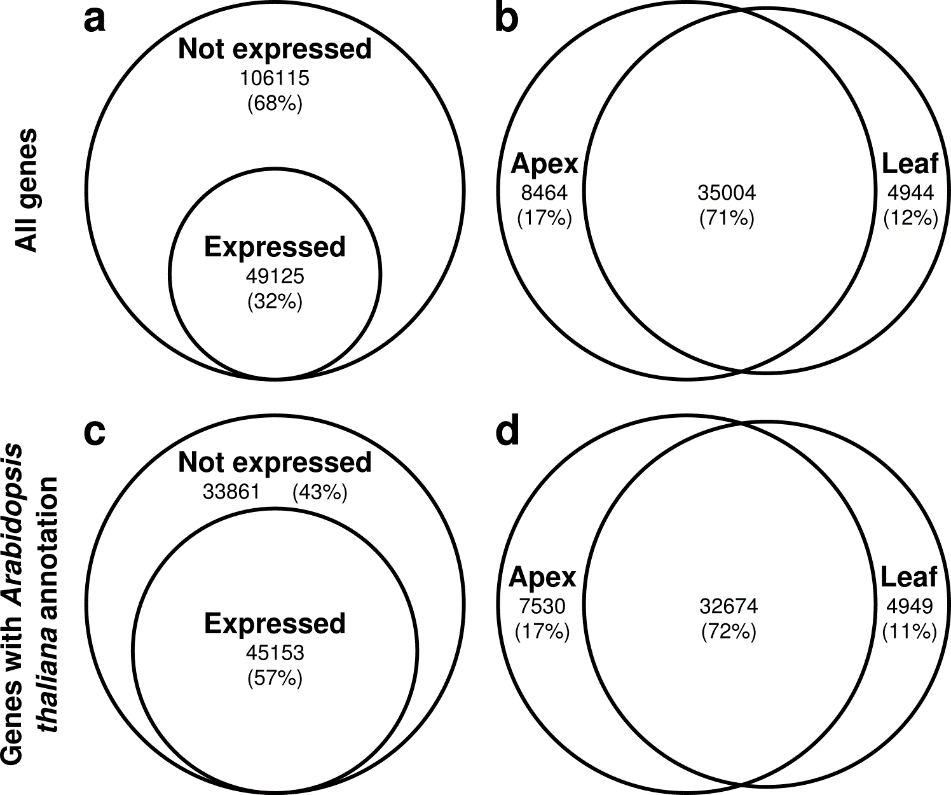
Supplementary figure 7 – Euler and Venn diagrams showing the percentage of expressed genes and the percentage of genes expressed in the apex and leaf sample**s**.

##
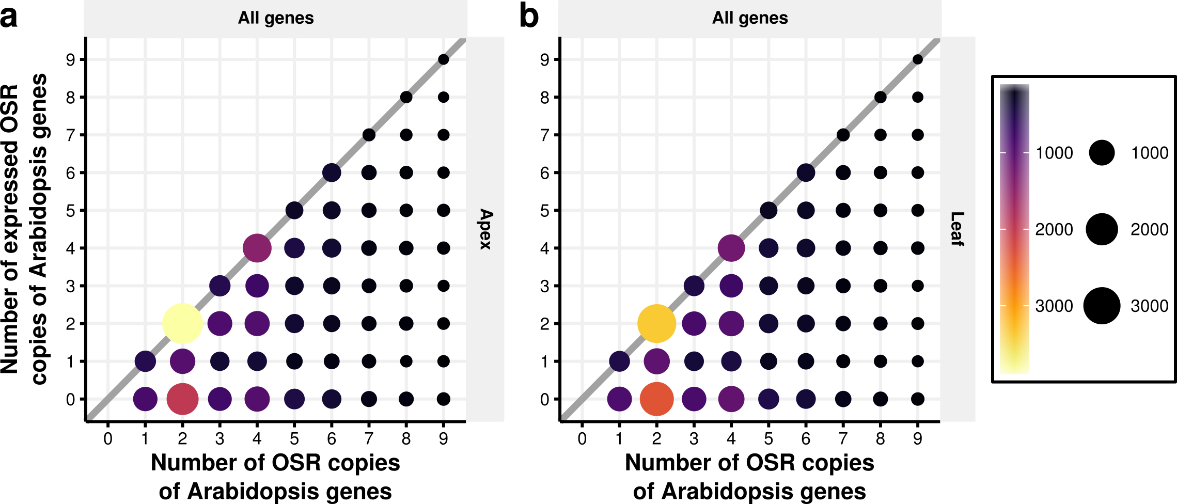
Supplementary figure 8 – Not all annotated OSR copies of Arabidopsis genes are expressed.

##
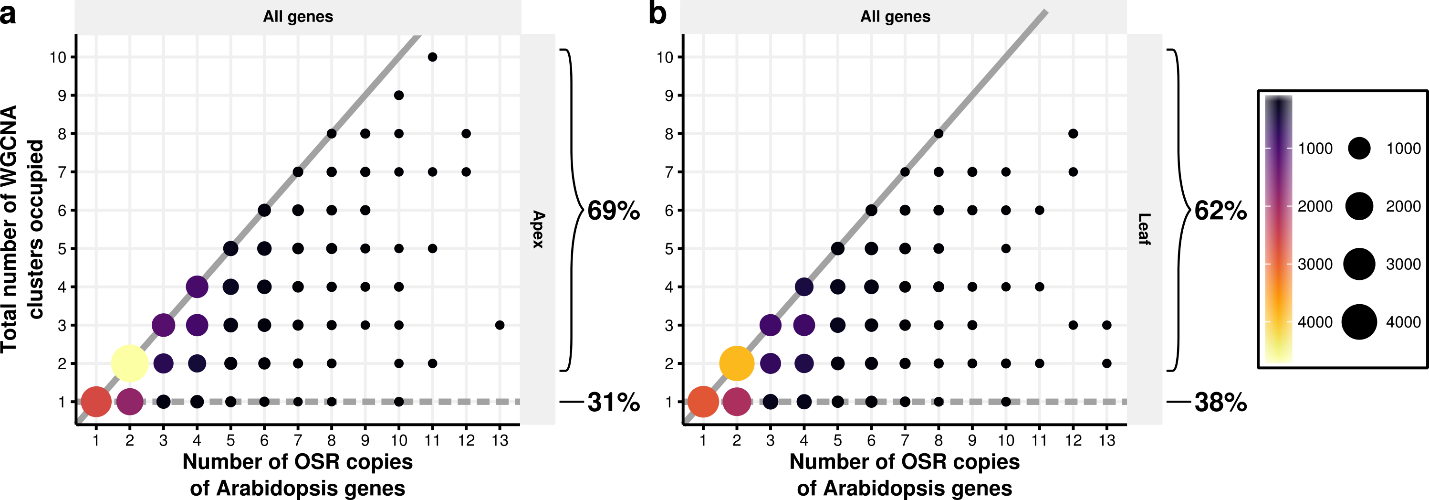
Supplementary figure 9 – Many gene copies are assigned to different regulatory modules in OSR*.*
