# Supplementary material for: Spatio‐temporal expression dynamics differ between homologues of flowering time genes in the allopolyploid Brassica napus
Source: Plant J. 2018 Aug 24;96(1):103–18. doi: 10.1111/tpj.14020 (PMC6175450; doi:10.1111/tpj.14020)
Supplement: Supplementary file 2 — Table S1. Sampling and sequencing scheme for the developmental time series. Table S2. Number of genes expressed two‐fold higher than their homologue for all flowering time gene homologue pairs. Table S3. Quantitative PCR primer sequences. Table S4. Sequencing statistics for the two sequencing runs carried out to generate the developmental transcriptome. [file TPJ-96-103-s002.docx]

| Date sampled | Days post sowing | Days vernalised | Days post vernalisation | Tissue Type | |
| --- | --- | --- | --- | --- | --- |
|  |  |  |  | Leaf | Apex |
| 2014-05-29 | 22 | 0 | - | 2 | 2 |
| 2014-06-19 | 43 | 21 | - | 2 | 2 |
| 2014-07-10 | 64 | 42 | - | 2 | 2 |
| 2014-07-11 | 65 | 42 | 1 | 1 | 1 |
| 2014-07-13 | 67 | 42 | 3 | 2 | 2 |
| 2014-07-15 | 69 | 42 | 5 | 0 | 1 |
| 2014-07-18 | 72 | 42 | 8 | 2 | 2 |

## Supplementary table 1 – Sampling and sequencing scheme for the developmental time series.

| Days post sowing | Apex | | |  | Leaf | | |
| --- | --- | --- | --- | --- | --- | --- | --- |
|  | Both expressed | A genome  2-fold higher | C genome  2-fold higher |  | Both expressed | A genome  2-fold higher | C genome  2-fold higher |
| 22 | 136 | 11 (8.1%) | 19 (14.0%) |  | 109 | 8 (7.3%) | 14 (12.8%) |
| 43 | 149 | 15 (10.1%) | 24 (16.1%) |  | 118 | 12 (10.2%) | 16 (13.6%) |
| 64 | 147 | 12 (8.2%) | 20 (13.6%) |  | 114 | 11 (9.6%) | 13 (11.4%) |
| 65 | 145 | 13 (9.0%) | 25 (17.2%) |  | 108 | 10 (9.3%) | 16 (14.8%) |
| 67 | 138 | 14 (10.1%) | 19 (13.8%) |  | 112 | 7 (6.3%) | 12 (10.7%) |
| 69 | 139 | 11 (7.9%) | 18 (12.9%) |  | - | - | - |
| 72 | 142 | 15 (10.6%) | 21 (14.8%) |  | 112 | 5 (4.5%) | 14 (12.5%) |

## Supplementary table 2 – Number of genes expressed 2-fold higher than their homoeologue for all flowering time gene homoeologue pairs.

| Gene | Forward Primer (5’ – 3’) | Reverse Primer (5’ – 3’) | Amplicon Length |
| --- | --- | --- | --- |
| *TFL1* A10 | GTCTCCAATGGCCATGAGT | GTGCCGGGGATGTTCATG | 179 |
| *TFL1* Cnn | GTCATGAACATCCCCGGC | GATCATTCTCGATCGCAAATTCA | 196 |
| *TFL1* C2 | CTGATGTTCCAGGTCCTAGC | TGGGGAGATATCGATAACATGTC | 197 |
| *TFL1* C3 | GAGGTGGTGAGCTATGAGTTG | CTGGGCGTTAAAGAAGACAGCA | 189 |
| *GAPDH* | AGAGCCGCTTCCTTCAACATCATT | TGGGAACACGGAAGGACATTCC | 112 |

## Supplementary table 3 – qPCR primer sequences.

| Tissue | Days post sowing | Sequencing Run 1 | | | |  | Sequencing Run 2 | | | |
| --- | --- | --- | --- | --- | --- | --- | --- | --- | --- | --- |
|  |  | Total reads (millions) | Mapped reads (millions / percentage of total) | Multiply mapping reads (millions / percentage of mapped) | Reads mapped to over 20 positions (ten thousand / percentage of mapped) |  | Total reads (millions) | Mapped reads (millions / percentage of total) | Multiply mapping reads (millions / percentage of mapped) | Reads mapped to over 20 positions (ten thousand / percentage of mapped) |
| Apex | 22 | 75.6 | 61.8 (81.8%) | 8.3 (13.4%) | 20.7 (0.3%) |  | 41.9 | 34.3 (81.9%) | 4.7 (13.8%) | 7.8 (0.2%) |
| Apex | 43 | 71.5 | 56.8 (79.4%) | 7.4 (13.1%) | 17.8 (0.3%) |  | 31.7 | 25.3 (79.8%) | 3.4 (13.6%) | 5.3 (0.2%) |
| Apex | 64 | 70.5 | 57.4 (81.4%) | 7.5 (13.0%) | 21.6 (0.4%) |  | 28.7 | 23.3 (81.2%) | 3.2 (13.8%) | 149.4 (6.4%) |
| Apex | 65 | 67.6 | 54.6 (80.7%) | 7.2 (13.2%) | 26.5 (0.5%) |  | NA | NA | NA | NA |
| Apex | 67 | 78.6 | 63.5 (80.8%) | 8.4 (13.2%) | 36.3 (0.6%) |  | 30.5 | 25.1 (82.3%) | 3.5 (13.9%) | 5.6 (0.2%) |
| Apex | 69 | 66.2 | 54.4 (82.2%) | 7.3 (13.5%) | 30.7 (0.6%) |  | NA | NA | NA | NA |
| Apex | 72 | 59.7 | 48.6 (81.4%) | 6.4 (13.2%) | 35.2 (0.7%) |  | 31.5 | 25.8 (81.8%) | 3.6 (14.1%) | 4.5 (0.2%) |
| Leaf | 22 | 68.2 | 54.7 (80.2%) | 8.4 (15.4%) | 9.5 (0.2%) |  | 33.9 | 28.0 (82.5%) | 4.4 (15.7%) | 3.7 (0.1%) |
| Leaf | 43 | 50.5 | 41.5 (82.1%) | 6.2 (15.0%) | 11.1 (0.3%) |  | 33 | 26.4 (80.1%) | 4.0 (15.1%) | 4.6 (0.2%) |
| Leaf | 64 | 73.9 | 60.7 (82.1%) | 8.8 (14.4%) | 10.2 (0.2%) |  | 35.5 | 29.1 (82.1%) | 4.3 (14.8%) | 3.7 (0.1%) |
| Leaf | 65 | 45.7 | 37.6 (82.2%) | 5.5 (14.6%) | 5.4 (0.1%) |  | NA | NA | NA | NA |
| Leaf | 67 | 81.8 | 67.1 (82.1%) | 10.0 (14.9%) | 9.4 (0.1%) |  | 35.7 | 28.8 (80.7%) | 4.4 (15.4%) | 3.5 (0.1%) |
| Leaf | 72 | 49 | 40.3 (82.1%) | 5.8 (14.5%) | 5.8 (0.1%) |  | 32.2 | 26.2 (81.2%) | 3.9 (15.1%) | 3.9 (0.1%) |

## Supplementary table 4 – Sequencing statistics for the two sequencing runs carried out to generate the developmental transcriptome.
