# Supplementary material for: Spatio‐temporal expression dynamics differ between homologues of flowering time genes in the allopolyploid Brassica napus
Source: Plant J. 2018 Aug 24;96(1):103–18. doi: 10.1111/tpj.14020 (PMC6175450; doi:10.1111/tpj.14020)
Supplement: Supplementary file 3 — Data S1. Supplementary results [file TPJ-96-103-s003.docx]

Supplementary Material

1. Spatio-temporal expression dynamics differ between flowering time gene homologues in the allopolyploid *Brassica napus*

D. Marc Jones*1,2*, Rachel Wells*1*, Nick Pullen*1*, Martin Trick*2*, Judith A. Irwin*1**, Richard J. Morris*1,2*^*^

^1^ Crop Genetics, John Innes Centre, Norwich Research Park, Norwich. NR4 7UH. United Kingdom.

^2^ Computational and Systems Biology, John Innes Centre, Norwich Research Park, Norwich. NR4 7UH. United Kingdom.

^*^Corresponding author: judith.irwin@jic.ac.uk

^*^Corresponding author: richard.morris@jic.ac.uk

Keywords: *Brassica napus*, gene duplication, flowering time, polyploid, regulation, time series

# Supplementary results

## A self-organising map based approach corroborates the finding that oilseed rape (*Brassica napus*; OSR) copies of *Arabidopsis thaliana* (Arabidopsis) flowering time genes have diverged in their regulation.

A self-organizing map (SOM) based approach was employed to detect expression divergence between OSR copies of flowering time genes. The advantage of this approach, over the WGCNA approach discussed in the main text, is that the expression module assignments are not binary, allowing for more subtle patterns to be detected. A SOM is a construct that groups together expression traces into clusters. The sampling procedure (Figure S2a) returns an empirical probability of two expression traces mapping to the same SOM cluster. In addition, clustering probabilities can be calculated for a single gene, which represent the uncertainty in the expression measurements quantified for that gene. In this case the clustering probability calculated is referred to as a self-clustering probability. Visualizing the clustering probabilities determined by the SOM based method is complicated by the bimodal distribution the probabilities follow. Figure S3 reveals a peak in self-clustering probabilities at 0.05 but also at ~1.0. This bimodal structure is a result of some genes only being expressed at a single time point. When these genes are resampled, their normalized expression traces remain the same, leading to a high self-clustering probability. To visualize probabilities from across this distribution, a soft threshold is applied to the probabilities. After the threshold is applied, the higher the clustering coefficient, the more similar two expression traces will tend to be. Genes are assigned to expression modules using heatmaps of clustering coefficients. The different patterns of regulatory module assignment are described in the main text.

This method was applied to OSR flowering time genes. The occurrences of the different expression module assignment patterns were counted for both apex (Figure S2b) and leaf (Figure S2c) expression data. The null hypothesis used in the WGCNA analysis was that copies of genes would not show divergence in their expression dynamics (dashed lines in Figure 7, main text). The *redundant* pattern in the SOM analysis is equivalent to this null hypothesis (Figure 8a, main text). Like the results from the WGCNA analysis, this null hypothesis is not true for any flowering time genes with five or more copies in the OSR leaf (Figure S2c) or six or more copies in the apex (Figure 2Sb). As with the *redundant* pattern, the *unique* pattern of expression module assignment becomes less frequent as the number of OSR copies of a gene increases (Figure S2b and S2c). This agrees with the WGNCA analysis, where the number of genes lying on the solid line in Figure 7 in the main text (equivalent to the *unique* pattern in the SOM analysis) decreases at higher numbers of copies.

WGCNA cannot detect *gradated* and *mixed* patterns of expression module assignment. In the apex and leaf, *mixed* and *gradated* patterns are seen at a lower frequency than *distinct* patterns, revealing that genes exhibiting intermediary expression dynamics relative to the other copies of that gene are observed less frequently than genes occupying distinct expression modules. The low number of *gradated* patterns observed when three genes copies are present in both tissues suggests that these genes tend to have expression traces that are detectably different to one another. *Distinct* patterns are more prevalent than *unique* patterns at three gene copies; revealing the majority contain one copy with an expression trace divergent to the expression traces of the other two copies.

We integrated homoeologue information for the three copy genes exhibiting a *distinct* pattern of expression module assignment to ask whether genes tended to be within the same expression modules as their homoeologue. In the apex, this is the case, with 59% of genes located in the same module. More generally, we find that of the genes in the apex (leaf) where homoeologue information is available, 69% (64%) of genes are assigned to the same module as the homoeologue, 18% (19%) of genes are assigned to a different module and 12% (16%) of genes have homoeologues which cannot be clustered. Homoeologues that cannot be clustered arise when the clustering coefficient calculated using the self-clustering probability of a gene is below 0.5, or the homoeologue is not expressed in that tissue.

We then asked whether the relatively large number of *distinct* patterns at four gene copies was due to homoeologous copies of genes displaying similar expression traces. For the genes for which homoeologue information was available, we find the majority (76% in apex, 72% in leaf) of genes are in the same expression module as their homoeologue.

The SOM analysis corroborates many of the key findings of the WGCNA analysis in a manner which takes into account the uncertainty in our data. Namely, that expression divergence between copies is widespread and that as the number of copies of a gene in the genome increases, the likelihood of observing regulatory divergence between those copies increases. Additionally, the SOM analysis reveals that some copies of flowering time genes exhibit a *gradated* pattern of expression module assignment, representing subtle differences in regulation. This may be the result of different subsets of regulatory proteins controlling the expression of the gene copies, or represents subtle functional differences that have consequences for the control of flowering time in OSR.

# References

Bouché, F., Lobet, G., Tocquin, P., and Périlleux, C. (2016). FLOR-ID: an interactive database of flowering-time gene networks in Arabidopsis thaliana. *Nucleic Acids Res.* 44, D1167–D1171. doi:10.1093/nar/gkv1054.

Chalhoub, B., Denoeud, F., Liu, S., Parkin, I. A. P., Tang, H., Wang, X., et al. (2014). Early allopolyploid evolution in the post-Neolithic Brassica napus oilseed genome. *Science* 345, 950–953. doi:10.1126/science.1253435.

Consortium, A. I. M. (2011). Evidence for Network Evolution in an Arabidopsis Interactome Map. *Science* 333, 601–607. doi:10.1126/science.1203877.

Kim, D., Pertea, G., Trapnell, C., Pimentel, H., Kelley, R., and Salzberg, S. L. (2013). TopHat2: accurate alignment of transcriptomes in the presence of insertions, deletions and gene fusions. *Genome Biol.* 14, R36. doi:10.1186/gb-2013-14-4-r36.

Lou, P., Wu, J., Cheng, F., Cressman, L. G., Wang, X., and McClung, C. R. (2012). Preferential Retention of Circadian Clock Genes during Diploidization following Whole Genome Triplication in Brassica rapa. *Plant Cell* 24, 2415–2426. doi:10.1105/tpc.112.099499.
